# Growth in chikungunya virus-related research in ASEAN and South Asian countries from 1967 to 2022 following disease emergence: a bibliometric and graphical analysis

**DOI:** 10.1186/s12992-023-00906-z

**Published:** 2023-02-06

**Authors:** Fajar Sofyantoro, Andri Frediansyah, Dwi Sendi Priyono, Wahyu Aristyaning Putri, Nur Indah Septriani, Nastiti Wijayanti, Winda Adipuri Ramadaningrum, Safaa A. Turkistani, Mohammed Garout, Mohammed Aljeldah, Basim R. Al Shammari, Ameen S. S. Alwashmi, Amal H. Alfaraj, Abdulsalam Alawfi, Amer Alshengeti, Maha H. Aljohani, Sahar Aldossary, Ali A. Rabaan

**Affiliations:** 1 Faculty of Biology, Universitas Gadjah Mada, Yogyakarta, 55281 Indonesia; 2 Center for Tropical Biodiversity, Faculty of Biology, Universitas Gadjah Mada, Yogyakarta, 55281 Indonesia; 3 PRTPP, National Research and Innovation Agency (BRIN), Yogyakarta, 55861 Indonesia; 4 National Agency of Drug and Food Control (Badan POM), Jakarta, 10560 Indonesia; 5 Fakeeh College for Medical Science, Jeddah, 21134 Saudi Arabia; 6 Department of Community Medicine and Health Care for Pilgrims, Faculty of Medicine, Umm Al-Qura University, Makkah, 21955 Saudi Arabia; 7 Department of Clinical Laboratory Sciences, College of Applied Medical Sciences, University of Hafr Al Batin, Hafr Al Batin, 39831 Saudi Arabia; 8 Department of Medical Laboratories, College of Applied Medical Sciences, Qassim University, Buraydah, 51452 Saudi Arabia; 9 Pediatric Department, Abqaiq General Hospital, First Eastern Health Cluster, Abqaiq, 33261 Saudi Arabia; 10 Department of Pediatrics, College of Medicine, Taibah University, Al-Madinah, 41491 Saudi Arabia; 11 Department of Infection Prevention and Control, Prince Mohammad Bin Abdulaziz Hospital, National Guard Health Affairs, Al-Madinah, 41491 Saudi Arabia; 12 Department of infectious diseases, King Fahad Hospital, Madinah, 42351 Saudi Arabia; 13 Pediatric Infectious Diseases, Women and Children’s Health Institute, Johns Hopkins Aramco Healthcare, Dhahran, 31311 Saudi Arabia; 14 Molecular Diagnostic Laboratory, Johns Hopkins Aramco Healthcare, Dhahran, 31311 Saudi Arabia; 15 College of Medicine, Alfaisal University, Riyadh, 11533 Saudi Arabia; 16 Department of Public Health and Nutrition, The University of Haripur, Haripur, 22610 Pakistan

**Keywords:** Chikungunya, ASEAN, South Asian, Arbovirus, Scopus, Bibliometric, VOSviewer

## Abstract

**Background:**

ASEAN (Association of Southeast Asian Nations) is composed of ten Southeast Asian countries bound by socio-cultural ties that promote regional peace and stability. South Asia, located in the southern subregion of Asia, includes nine countries sharing similarities in geographical and ethno-cultural factors. Chikungunya is one of the most significant problems in Southeast and South Asian countries. Much of the current chikungunya epidemic in Southeast Asia is caused by the emergence of a virus strain that originated in Africa and spread to Southeast Asia. Meanwhile, in South Asia, three confirmed lineages are in circulation. Given the positive correlation between research activity and the improvement of the clinical framework of biomedical research, this article aimed to examine the growth of chikungunya virus-related research in ASEAN and South Asian countries.

**Methods:**

The Scopus database was used for this bibliometric analysis. The retrieved publications were subjected to a number of analyses, including those for the most prolific countries, journals, authors, institutions, and articles. Co-occurrence mapping of terms and keywords was used to determine the current state, emerging topics, and future prospects of chikungunya virus-related research. Bibliometrix and VOSviewer were used to analyze the data and visualize the collaboration network mapping.

**Results:**

The Scopus search engine identified 1280 chikungunya-related documents published by ASEAN and South Asian countries between 1967 and 2022. According to our findings, India was the most productive country in South Asia, and Thailand was the most productive country in Southeast Asia. In the early stages of the study, researchers investigated the vectors and outbreaks of the chikungunya virus. In recent years, the development of antivirus agents has emerged as a prominent topic.

**Conclusions:**

Our study is the first to present the growth of chikungunya virus-related research in ASEAN and South Asian countries from 1967 to 2022. In this study, the evaluation of the comprehensive profile of research on chikungunya can serve as a guide for future studies. In addition, a bibliometric analysis may serve as a resource for healthcare policymakers.

## Background

Chikungunya virus, which was first isolated in Tanzania in 1953 [1, 2], has been identified in Asia, the Americas, the Pacific Islands, Europe, and Africa [3–8]. Chikungunya is part of the arbovirus (arthropod-borne) group, and it is considered the source of Chikungunya fever, an acute febrile sickness inciting millions of mortality cases worldwide [9–11]. The term “chikungunya”, which originated from the Makonde language of Tanzania, is attributed to the “bending posture” of patients infected with the chikungunya virus who experience excruciating pain in the joints, which might persist for months [1, 12–14]. Apart from severe muscular and joint pain, chikungunya virus infections are also accompanied by headaches, rashes, photophobia, and fever [15–18]. Prolonged chikungunya virus infection may trigger adverse effects, including brain inflammation, damage to optic nerves, injury to the spinal cord, Guillain–Barré syndrome, heart muscle inflammation, hepatitis, a renal lesion, or even death [19–24].

The transmission routes of the chikungunya virus mostly occur via urban and sylvatic cycles [25]. The sylvatic routes involve various types of organisms, including several *Aedes* species and non-human primate species, such as monkeys, baboons, and mandrills [25–30]. In recent years, *Culex* and *Anopheles* have also been recognized as vectors of the chikungunya virus [28]. Transmission via the urban route primarily occurs via *Aedes albopictus* and *Aedes aegypti*, which are reported as the primary vectors of chikungunya virus in Asia, Oceania, Europe, the Americas, and Africa [31–36]. Notably, *A. aegypti* also transmits other types of viruses, including dengue virus and zika virus, as supported by several studies reporting the occurrence of dengue/chikungunya/zika virus or dengue/chikungunya virus simultaneous infections [37–39]. At present, specific antiviral drugs for the chikungunya virus are not available; thus, treatment of the chikungunya virus-infected patients focuses on treating the observed symptoms [40, 41]. Recent developments in therapeutic strategies for treating chikungunya virus infection focus on antiviral drugs inhibiting viral adsorption, protein translation, genome replication, glycoprotein maturation, and activation of the immune system [42–48].

The primary goals of ASEAN, also known as the Association of Southeast Asian Nations, are to foster regional stability and economic progress among its members [49]. ASEAN consisted of ten nations, including Vietnam, Thailand, Singapore, the Philippines, Myanmar, Malaysia, Laos, Cambodia, Brunei Darussalam, and Indonesia. In recent years, the consumption of animal-based foods has rapidly increased in the Southeast Asia region, as a result of massive urbanization, rising incomes, and the expansion of industrialization [50, 51]. As a consequence, the Southeast Asian countries are prone to outbreaks of epizootic and zoonotic diseases [52, 53]. Threats to animal and human health are serious, since novel disease outbreaks have the potential to spread quickly across borders and may result in major socio-economic and public health casualties [54]. In addition, as a result of natural disasters, such as earthquakes and extreme weather, the Southeast Asia region might face food safety and public health crises [55, 56]. A number of zoonotic disease outbreaks in ASEAN countries, including chikungunya, caused serious consequences, including human deaths and economic losses [57–60]. In addition, significant numbers of chikungunya cases have been reported in Singapore, Malaysia, and Thailand [59]. Therefore, the shared vulnerability between ASEAN countries emphasizes the necessity of a collective response. By anticipating risks and taking prompt action in response to possible threats, the public and animal health systems in ASEAN countries will be more resilient and sustainable.

Among South Asian countries, India is estimated to contain the highest number of chikungunya virus infection cases [7, 61–63]. In addition, Bangladesh, Bhutan, Pakistan, Sri Lanka, Nepal, and the Maldives have also reported outbreaks of chikungunya [7, 62–64]. Multiple chikungunya virus lineages are confirmed to be circulating in South Asia, including the East-Central-South Africa (ECSA), Asian, and Indian Ocean lineages [62, 65–67]. The ECSA, which originated in Africa, has spread to South Asia and become predominant in recent years [63]. The ECSA lineage also gives rise to another unique lineage known as the Indian Ocean Lineage (IOL) [18, 65, 68, 69]. Meanwhile, the Asian Lineage (AL), another lineage detected in South Asia, was originally discovered during epidemics in Asian countries between 1958 and 1973 [62, 65]. South Asian countries are prone to chikungunya virus outbreaks and re-emergences. Because of the cyclical nature of chikungunya virus infection, outbreaks in endemic regions, such as South Asian countries, reoccur every few years. Furthermore, the persistent epidemics of chikungunya virus in other parts of the world, particularly Africa, have a substantial impact on its potential re-emergence in South Asian countries. Another urgent and serious concern for South Asian countries is the high probability of dengue and chikungunya viruses co-circulation [70, 71].

Bibliometric methodology has been established as a primary tool to evaluate research performance in various fields, including health [72, 73], sciences [74, 75], socials [76, 77], toxicology [78, 79], engineering [80, 81], and environmental studies [82, 83]. Nonetheless, bibliometric studies on chikungunya are limited [84, 85]. At present, chikungunya-related studies are receiving considerable attention from international scientific communities because of several outbreaks that have happened in recent years [63, 86–92]. Thus, the prevailing chikungunya-related literature from various perspectives must be evaluated and categorized thoroughly. The current study aims to adopt bibliometric techniques for assessing the scientific output of chikungunya-related studies by ASEAN and South Asian countries and identify areas of concern for future research. In the current study, the evaluation of chikungunya-related articles included authors, journals, institutions, countries of origin, and citation analysis. Identifying relevant topics and emerging trends might help researchers in ASEAN and South Asian countries working in the field of chikungunya. The findings in our study are also applicable to public leaders who strive to make well-informed decisions based on evidence-based policymaking.

## Methods

**Fig. 1:**
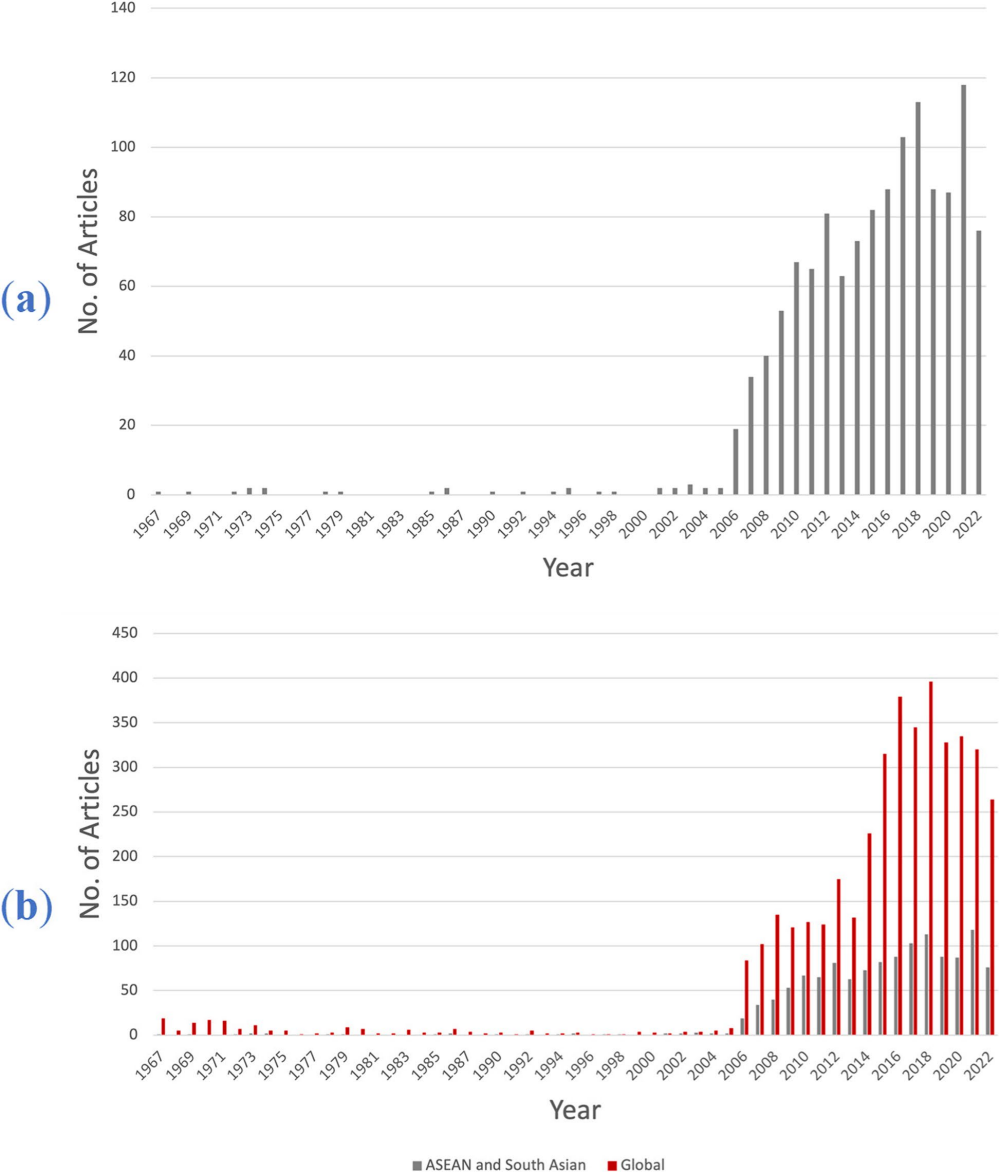
Trend of published articles on the chikungunya virus from 1967 to 2022 by ASEAN and South Asian countries. **a** Annual productivity of ASEAN and South Asian countries, totaling in1280 documents; **b** Contribution of ASEAN and South Asian countries to global productivity

Documents associated with the chikungunya virus from ASEAN and South Asian countries indexed in Scopus from 1967 to 2022, excluding erratum and retracted articles, were extracted for our bibliometric study. To avoid including duplicate or invalid documents in the analysis, erratum and retracted articles were excluded. In this study, the Scopus database served as our sole data source because it covered a large range of journals and a high number of articles relative to other libraries such as Web of Science or PubMed [93, 94]. Scopus has also been validated and recognized in other bibliometric analyses [75, 79, 95–97]. Data extraction was performed on December 17, 2022. The key term “chikungunya” was registered to identify chikungunya-related articles in Scopus. The search strategy for “chikungunya” was limited to titles only to improve accuracy. If additional search fields such as abstract or keywords were included, most of the extracted articles were false-positive data and not directly linked to chikungunya. Additional filtering was also established to exclusively retrieve documents published by ASEAN countries (Brunei Darussalam, Cambodia, Indonesia, Laos, Malaysia, Myanmar, the Philippines, Singapore, Thailand, and Vietnam) and South Asian countries (Afghanistan, Bangladesh, Bhutan, the British Indian Ocean Territory, India, the Maldives, Nepal, Pakistan, and Sri Lanka). The following parameters were considered during the assessment of the extracted articles: the number of published articles per year, countries with prolific publication profiles, influential journals, institutions, and most cited articles. Based on the Scopus algorithm, countries and institutions were extracted from all authors associated with the published articles, regardless of the sequence or the position of the authors. Therefore, an article might be linked to several countries or institutions. In the current study, the global profile of chikungunya virus-related literature was extracted as well, irrespective of the countries of origin. The global profile data was used to measure the contribution of ASEAN and South Asian countries (Fig. 1b), and to calculate the global ranks of countries and institutions (Tables 2 and 4). Bibliometrix was used to analyze the data, and VOSviewer was used to construct maps of visual networks projecting collaborations and trending research topics [98, 99].

## Results

From 1967 to 2022, the Scopus algorithm identified 1280 documents published by ASEAN and South Asian countries in the chikungunya-related field, accounting for 31.11% of the global productivity (*n* = 4107). Our search results, focusing on ASEAN and South Asian countries, included 1011 (78.98%) articles, 103 (8.04%) letters, 92 (7.18%) reviews, 20 (1.56%) notes, 17 (1.32%) conference papers, and 39 (3.04%) other types of documents, including book chapters, editorials, notes, and short surveys. Among the published documents, English is the most common language, accounting for 1279 (99.92%) of the total documents.

**Fig. 2:**
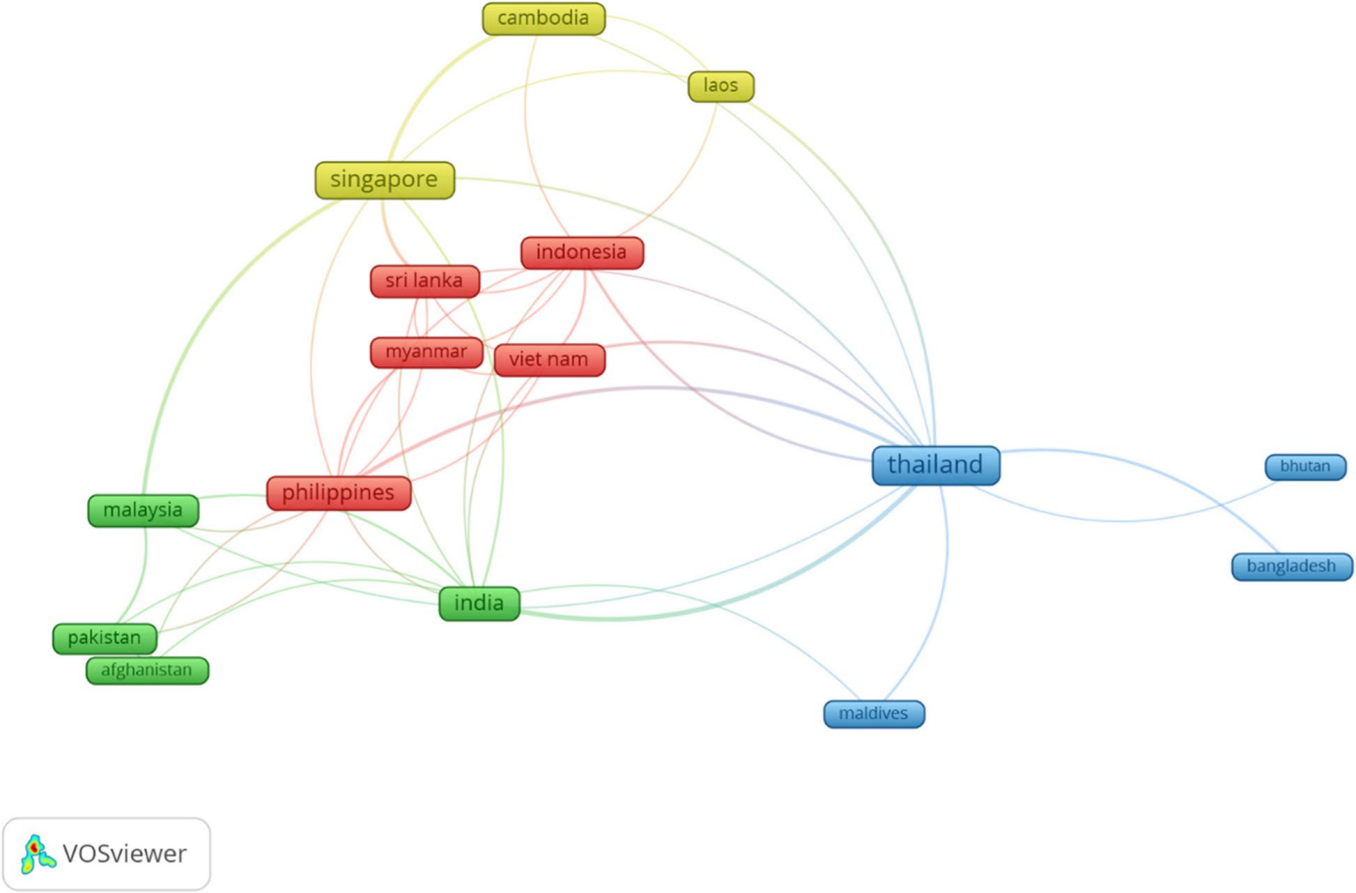
Mapping of country collaboration between ASEAN and South Asian countries. Countries assigned larger frames represent a relatively higher number of collaborations

The chikungunya virus publishing trend in ASEAN and South Asian countries exhibited a gradual increase from 1967 to 2022 (Fig. 1a). In addition, ASEAN and South Asian countries showed a significant contribution to the global productivity of chikungunya virus-related publications (Fig. 1b). Fig. 2 displays the visualization of collaboration among ASEAN and South Asian countries. The relative size of the frames represents the number of collaborations. For example, the frame size of Thailand is relatively larger than that of the Maldives, indicating that Thailand (*n* = 12) builds collaborative studies with more countries compared to the Maldives (*n* = 2).

The productivity among ASEAN and South Asian countries in research documents related to the chikungunya virus was also ranked, with India having the highest percentage (Table 1). The top ten journals publishing articles related to the chikungunya virus produced by ASEAN and South Asian countries were also listed (Table 2). The most documents were published in PLoS Neglected Tropical Diseases (*n* = 46; 3.59%), followed by PLoS ONE (*n* = 36; 2.81%), and the Indian Journal of Medical Research (*n* = 33; 2.57%). Also, the characteristics of papers published by ASEAN and South Asian countries with the highest number of citations in the last five decades were summarized (Table 3) [100–109].

**Table 1:** Top ten productive ASEAN and South Asian countries in the field of chikungunya

No.	Country	No. of Articles (%)	Global Rank
1	India	743 (58.04)	2
2	Thailand	151 (11.79)	7
3	Singapore	134 (10.46)	10
4	Malaysia	88 (6.87)	14
5	Pakistan	54 (4.21)	22
6	Bangladesh	46 (3.59)	23
7	Indonesia	38 (2.96)	26
8	Sri Lanka	24 (1.87)	37
9^a^	The Phillipines	15 (1.17)	49^a^
9^a^	Vietnam	15 (1.17)	49^a^

^a^Countries with the same number of articles are listed as having the same rank

**Table 2:** Journals publishing articles related to the chikungunya virus produced by ASEAN and South Asian countries

No.	Journal Title	No. of Articles (%)	Impact Factor^**a**^	SNIP^**b**^
1	PLoS Neglected Tropical Diseases	46 (3.59)	4.41	1.640
2	PLoS ONE	36 (2.81)	3.24	1.368
3	Indian Journal of Medical Research	33 (2.57)	2.37	
4	American Journal of Tropical Medicine and Hygiene	30 (2.34)	2.34	1.090
5	Scientific Reports	25 (1.95)	4.37	1.389
6	Emerging Infectious Diseases	24 (1.87)	6.88	2.771
7	Transactions of the Royal Society of Tropical Medicine and Hygiene	21 (1.64)	–	0.801
8^c^	Antiviral Research	18 (1.40)	5.97	1.680
8^c^	Journal of Medical Virology	18 (1.40)	2.32	2.756
8^c^	Virology Journal	18 (1.40)	4.09	1.307

^a^Impact factors (IF), as reported in Journal Citation Reports (JCR) 2021 of Clarivate Analytics. ^b^SNIP (Source Normalized Impact per Paper) 2021, obtained from Scopus at www.scopus.com/sources

^c^Journals with the same number of articles are listed with the same rank

**Table 3:** Most cited chikungunya virus-related papers published by ASEAN and South Asian countries

No.	Authors	Title	No. of Citations	Journal Title	Year	Document Type
1	Pialoux et al. [100]	Chikungunya, an epidemic arbovirosis	767	Lancet Infectious Diseases	2007	Review
2	Musso et al. [101]	Zika virus: Following the path of dengue and chikungunya?	346	The Lancet	2015	Letter
3	Yergolkar et al. [102]	Chikungunya outbreaks caused by African genotype, India	266	Emerging Infectious Diseases	2006	Article
4	Chow et al. [103]	Persistent arthralgia induced by Chikungunya virus infection is associated with interleukin-6 and granulocyte macrophage colony-stimulating factor	254	Journal of Infectious Diseases	2011	Article
5	Arankalle et al. [104]	Genetic divergence of Chikungunya viruses in India (1963–2006) with special reference to the 2005–2006 explosive epidemic	253	Journal of General Virology	2007	Article
6	Ng et al. [105]	IL-1β, IL-6, and RANTES as biomarkers of Chikungunya severity	218	PLoS ONE	2009	Article
7	Burt et al. [106]	Chikungunya virus: an update on the biology and pathogenesis of this emerging pathogen	208	The Lancet Infectious Diseases	2017	Review
8	Laras et al. [107]	Tracking the re-emergence of epidemic chikungunya virus in Indonesia	205	Transactions of the Royal Society of Tropical Medicine and Hygiene	2005	Article
9	Her et al. [108]	Active infection of human blood monocytes by Chikungunya virus triggers an innate immune response	199	Journal of Immunology	2010	Article
10	Mavalankar et al. [109]	Increased mortality rate associated with chikungunya epidemic, Ahmedabad, India	181	Emerging Infectious Diseases	2008	Article

**Fig. 3:**
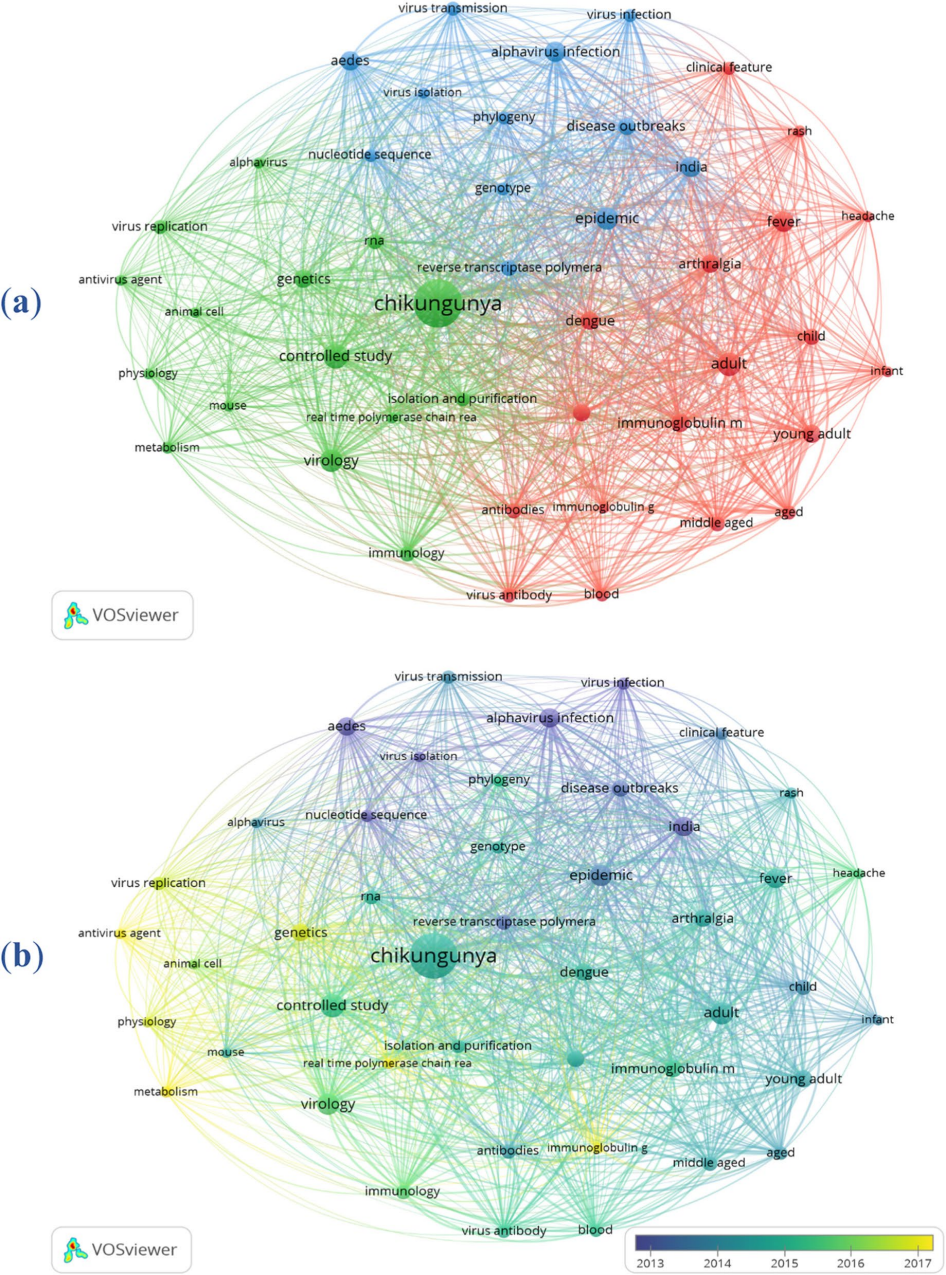
Co-occurrence network of terms extracted from articles published by ASEAN and South Asian countries in chikungunya virus-related articles from 1967 to 2022. The minimum number of occurrences was set to 100 times. Of the 7810 terms, 56 were included. **a** Network visualization; **b** Overlay visualization. Terms highlighted in blue appeared earlier than those assigned in yellow

A network visualization of terms used by authors from ASEAN and South Asian countries in the 55-year period of chikungunya virus-related research was constructed (Fig. 3a). The extracted papers yielded a total of 7810 different terms, 56 of which occurred more than 100 times. The main three central clusters are assessed as follows: cluster 1 (in red) primarily consists of terms related to “dengue,” “arthralgia,” “immunoglobulin”; cluster 2 (in green) includes “genetics,” “virology,” “isolation and purification”; and cluster 3 (in blue) highlights the terms “epidemics,” “disease outbreaks,” “alphavirus infection”. VOSviewer also assigned colors to chikungunya-related terms based on publication years (Fig. 3b). Purple indicates that the terms appear in the early years, whereas the yellow color is designated for terms that emerge in recent years. The progress of research on Chikungunya virus shows a dynamic change over time, with a focus on “*Aedes”,* “disease outbreaks,” and “alphavirus infection” in 2013; “epidemic,” “reverse transcriptase polymerase,” and “antibodies” in 2014; “isolation and purification,” “arthralgia,” and “dengue” in 2015. In 2016, research on “virology,” “animal cells,” “mice,” and “immunology” gained a significant attention in the field of chikungunya studies. Importantly, from 2017 onwards, the published articles mainly reported on “virus replication,” “metabolism,” “physiology,” “antivirus agent,” and “genetics” chikungunya virus, indicating that these terms are emerging and gaining significant attention in the last few years. The institutions from ASEAN and South Asian countries with the highest productivity in the field of chikungunya from 1967 to 2022 were also listed, highlighting the prominent contributions of India, Singapore, and Thailand (Table 4).

**Table 4:** Top ten institutions from ASEAN and South Asian countries with the highest performance in the field of chikungunya virus

No.	Institutions	Country	No. of Articles (%)	Global Rank
1	National Institute of Virology	India	79 (6.17)	8
2	Indian Council of Medical Research	India	66 (5.15)	10
3	National University of Singapore (NUS)	Singapore	65 (5.07)	11
4^a^	Mahidol University	Thailand	61 (4.76)	13^a^
4^a^	Agency for Science, Technology, and Research (A*STAR)	Singapore	61 (4.76)	13^a^
5	NUS Yong Loo Lin School of Medicine	Singapore	57 (4.45)	16
6	Universiti Malaya	Malaysia	47 (3.67)	25
7	Defence Research & Development Establishment (DRDE)	India	42 (3.28)	34
8	Chulalongkorn University	Thailand	33 (2.57)	45
9	Prince of Songkla University	Thailand	27 (2.10)	60

^a^Institutions with the same number of articles are listed as having the same rank

## Discussion

Our research provided new perspectives on the scientific contribution of ASEAN and South Asian countries to chikungunya virus-related research. In general, ASEAN and South Asian countries contribute 31.11% of the global production of chikungunya virus-related studies. In 2006, the number of articles published by ASEAN and South Asian countries increased dramatically, from 2 documents in 2005 to 19 documents in 2006. After 2006, a gradual increase in chikungunya-related documents produced by ASEAN countries was observed, indicating that chikungunya virus-related topics are gaining prominence as a human health concern. A closer examination of the documents published in 2006 reveals that India, with 15 documents, contributed the most articles. Articles published in 2006 mostly discussed the re-emergence of the chikungunya virus in India and Malaysia [102, 110]. Meanwhile, in 2009, Tan Tock Seng Hospital, Singapore, and the Agency for Science, Technology and Research, Singapore were among the numerous organizations that reported the epidemiology of a chikungunya outbreak in Singapore [60, 111, 112]. Notably, 2021 has the highest productivity for ASEAN and South Asian countries, with 118 (9.21%) documents published. Consistent with the overall results shown in Table 1, India ranked first in 2021 with the highest output, publishing 63 (4.92%) documents. In accordance with other bibliometric studies analyzing the research productivity of ASEAN and South Asian countries [113–116], the current study also highlighted the prominent contributions of India, Thailand, and Singapore in scientific research. Recently, consecutive chikungunya epidemics have occurred in developing countries [60, 63, 86, 87, 89, 111, 112, 117–120], which may also explain the increasing number of ASEAN and South Asian countries participating in chikungunya-related studies.

In bibliometric analysis, directly evaluating the weight or effect of publications is difficult. However, a number of studies argued that important insights could be obtained by analyzing the relationship between article relevancy and journal significance [121, 122]. In addition, the top ten most cited articles may provide hints into the current trends, the changing landscape of research topics, and important directions for future research, as shown in Table 3.

After 2017, the topics of antiviral activity and neutralizing antibodies are emerging as the new focus on chikungunya-related studies in ASEAN and South Asian countries. Effective antiviral strategies are required to combat the rising prevalence of chikungunya virus infection and reduce mortality. Because there are no effective vaccines, significant research has been conducted to discover potent antivirals against the chikungunya virus (Table 5). In parallel, repurposing currently available drugs to treat chikungunya infections has been proposed and intensively investigated as an alternative [42, 43, 46, 123–129]. Determining novel compounds with anti-chikungunya virus properties has also been a focus of research in recent years [47, 130–143]. Additionally, in silico methods have been employed to find promising molecules against chikungunya virus [144–153].

**Table 5:** Recent advances in the development of drugs for alternative treatment of chikungunya

**Repurposing commercially available drugs**			
**No.**	**Drugs**	**Original purpose/disease target**	**References**
1	Chloroquine	malaria	[42, 43, 123, 124]
2	Arbidol	influenza	[125, 126]
3	Imipramine	antidepressant drug	[46]
4	Ribavirin	Respiratory syncytial virus and hepatitis c virus	[127–129]
**Novel compounds with antiviral properties**			
**No.**	**Targeted pathway or process**	**Compounds**	**References**
1	Entry and binding	Flavagline FL3, FL23, and sulfonyl amidine 1 m	[47]
2		Curcumin	[130]
3	Replication	Andrographolide	[131]
4		Mycophenolic acid (MPA)	[132]
5		6-azauridine	[133]
6		Suramin	[134]
7		Harringtonine	[135]
8		debromoaplysiatoxin and 3-methoxydebromoaplysiatoxin	[136]
9		Phorbol-12, 13-didecanoate	[137]
10		Salicylate-derived Bryostatin analogues	[138]
11		Geldanamycin	[139]
12		Jatrophane ester	[140]
13		Trigocherrins A B, and F	[141]
14		MBZM-N-IBT	[142]
15		Abamectin, ivermectin, and berberine	[143]
**Promising molecules detected via in silico molecular docking**			
**No.**	**Protein targets**	**Compounds**	**References**
1	Non-structural Protein 2 (nsP2)	Astragaloside II-IV	[144]
2		ASN 01107557 and ASN 01541696	[145]
3		CID_5808891	[146]
4	Structural protein E3	Arjungenin	[147]
5	Non-structural Protein 3 (nsP3)	CMPD178	[148]
6		Hesperetin	[149]
7		Baicalin, Rutaecarpine, Amentoflavone, Apigetrin, Luteoloside, and Baloxavir	[150]
8		Baicalin	[151]
9	Non-structural Protein 4 (nsP4)	Mitoxantrone hydrochloride	[152]
10		LabMol-309	[153]
11	Capsid protein	Catechin-5-O-gallate and Rosmarinic acid	[147]

The majority of global efforts have been devoted to concentrating on treating symptoms and preventive measures, because there are no commercially available vaccines or medications to cure chikungunya infections [15]. The mitigation of mosquito bites, vector control, and disease containment are among the priorities of prevention efforts [154]. Meanwhile, to treat the infections, there are several pharmacologic and non-pharmacologic approaches have been developed. Systematic changes in lifestyle such as dietary adjustments, adequate fluid consumption, physiotherapy, and enough bed rest are examples of non-pharmacologic interventions, that are essential to supporting the work of the immune system in combating the virus [154–156]. On the other hand, pharmacologic therapies use medications to treat symptoms, such as joint pain and fever [106, 157]. Webb et al. reviewed the global Clinical Management Guidelines (CMGs) for chikungunya infection, reporting a lack of consistency in the classification of disease stages [158]. They also highlighted a variation in the prescriptions of corticosteroids and Nonsteroidal Anti-Inflammatory Drugs (NSAIDs) [158], which are essential in symptomatic treatment for individuals infected with the chikungunya virus [159]. Despite having a low risk of mortality, the chikungunya virus can be lethal for children and the elderly [160, 161]. The current CMGs for these vulnerable population groups, on the other hand, were limited and varied [158]. Transmission of the chikungunya virus could also be prevented by cutting the reproductive cycles of the vectors, *A. aegypti* and *A. albopictus* [154, 162]. Effective interventions include using larvicides, removing larval breeding sites, and reducing contact between humans and vectors [154, 163, 164]. Additionally, special care for infected individuals is critical part of the preventive measures, since they may contribute to the cycle of transmission.

This study uses a bibliometric technique to assess the current state and trajectory of chikungunya virus-related research published by ASEAN and South Asian countries. However, a few limitations, which also appear in other bibliometric analyses, were identified. First, our study registered the key term “chikungunya” only for title searches. Our analysis may have overlooked any publications that utilized the keyword “chikungunya” in the abstract or within the publication. Second, this study focused on publications indexed in the Scopus library. Although Scopus is the most widely used and trusted database, a few outlier papers may have been left out. Despite these limitations, our bibliometric study is the first to provide a concise overview of the chikungunya virus-related research profile by ASEAN and South Asian countries. Our research also demonstrates how bibliometric analysis may be used to estimate research productivity in the field of chikungunya.

## Conclusions

Our study is the first to present the contribution of ASEAN and South Asian countries to chikungunya virus-related research. By integrating literature review and bibliometric analysis, the current study aimed to provide an outline of the progress, current trend, and emerging topics in the field of chikungunya in ASEAN and South Asian countries. Over the past two decades, the research productivity on chikungunya virus-related topics in ASEAN and South Asian countries has increased remarkably, with a total of 1280 articles published in prominent journals. Previously, researchers prioritized studies related to the vector, infection, and nucleotide sequence of the chikungunya virus. However, in recent years, the research topics have shifted to the development of antiviral drugs and the genetics of chikungunya virus. Out of ten ASEAN countries, Thailand contributes the highest number of articles related to chikungunya, followed by Singapore. Meanwhile, India ranks first among South Asian countries in terms of productivity. The findings of this study can serve as a reference for ongoing chikungunya virus-related research and policymakers working in the field of healthcare, particularly in ASEAN and South Asian countries.

## Data Availability

All data generated or analyzed during this study are included in this published article. Other datasets used during the current study are available from the corresponding authors on reasonable request.
